# Patient-Specific Variations in Local Strain Patterns on the Surface of a Trussed Titanium Interbody Cage

**DOI:** 10.3389/fbioe.2021.750246

**Published:** 2022-01-11

**Authors:** Arjan C. Y. Loenen, Jérôme Noailly, Keita Ito, Paul C. Willems, Jacobus J. Arts, Bert van Rietbergen

**Affiliations:** ^1^ Laboratory for Experimental Orthopaedics, Department of Orthopaedic Surgery, CAPHRI, Maastricht University Medical Center, Maastricht, Netherlands; ^2^ Orthopaedic Biomechanics, Department of Biomedical Engineering, Eindhoven University of Technology, Eindhoven, Netherlands; ^3^ Department of Information and Communication Technologies, BCN MedTech, Universitat Pompeu Fabra, Barcelona, Spain

**Keywords:** low back pain, interbody fusion, finite element analysis, patient-specific, trussed titanium cage, bone mechanobiology, strain

## Abstract

**Introduction:** 3D printed trussed titanium interbody cages may deliver bone stimulating mechanobiological strains to cells attached at their surface. The exact size and distribution of these strains may depend on patient-specific factors, but the influence of these factors remains unknown. Therefore, this study aimed to determine patient-specific variations in local strain patterns on the surface of a trussed titanium interbody fusion cage.

**Materials and Methods:** Four patients eligible for spinal fusion surgery with the same cage size were selected from a larger database. For these cases, patient-specific finite element models of the lumbar spine including the same trussed titanium cage were made. Functional dynamics of the non-operated lumbar spinal segments, as well as local cage strains and caudal endplate stresses at the operated segment, were evaluated under physiological extension/flexion movement of the lumbar spine.

**Results:** All patient-specific models revealed physiologically realistic functional dynamics of the operated spine. In all patients, approximately 30% of the total cage surface experienced strain values relevant for preserving bone homeostasis and stimulating bone formation. Mean caudal endplate contact pressures varied up to 10 MPa. Both surface strains and endplate contact pressures varied more between loading conditions than between patients.

**Conclusions:** This study demonstrates the applicability of patient-specific finite element models to quantify the impact of patient-specific factors such as bone density, degenerative state of the spine, and spinal curvature on interbody cage loading. In the future, the same framework might be further developed in order to establish a pipeline for interbody cage design optimizations.

## Introduction

Lumbar interbody fusion (LIF) is a well-accepted treatment for low back pain symptoms that emerge from segmental mechanical instability (Fritzell et al., 2001; Bhalla et al., 2017). During LIF surgery, the intervertebral disc (IVD) of the affected segment is replaced by an interbody fusion cage. Interbody cages provide immediate mechanical support and serve as scaffold to facilitate bone growth in the intervertebral space and fuse the two adjacent vertebrae (Bagby, 1988). Although cages are usually enriched with bone graft (substitute) to foster bone formation (Duarte et al., 2017), both material and design of the inserted cage dominate the mechanical interplay and define the initial interface between host tissue and cage. Current interbody fusion cages still render suboptimal fusion rates following LIF treatment (Meng et al., 2021). For this reason, novel interbody cages are still being developed and introduced into the clinic.

One specific technique utilized to manufacture a new generation of interbody cages is metal additive manufacturing (Arts et al., 2020), commonly known as 3D printing. It builds an object layer-by-layer by selectively adding material where needed, thus enabling production of tailored porous implant designs that are biomechanically optimized (Tan et al., 2017; Pobloth et al., 2018). Examples of such novel 3D printed metal interbody cages are trussed titanium interbody fusion cages (Hunt et al., 2021). Trussed cages encompass a network of linear beam elements (struts) that join at several intersections within the design. These highly porous cages provide an open architecture to accommodate bone ingrowth and may deliver bone stimulating mechanobiological strains to the cells attached to the strut surfaces.

Previous *ex vivo* research quantified the strain in all the struts of a trussed cage under moderate (1,000 N) and strenuous (2,000 N) axial compressive loads, by using high resolution micro computed tomography (CT) imaging (Caffrey et al., 2016). Assuming that strain amplitudes over 200 µε (microstrain, 10^−6^ strain) are relevant to both preserve bone homeostasis and stimulate bone formation (Duncan and Turner, 1995), it was concluded that physiological loading of the cages induced strut strains consistent with those reported to maintain bone balance. Accordingly, it was demonstrated that cage design (e.g. diameter of struts) could be adjusted in order to tailor the strains induced by physiological mechanical loads (Caffrey et al., 2018).

Although the aforementioned *ex vivo* investigations provide valuable insights into the size and distribution of strut strains under physiological loading conditions and allow to explore design modifications, the experimental set-up entailed several limitations. Firstly, loading protocols were limited to static axial compression to allow for microCT image analysis. Secondly, strain magnitudes were quantified per strut, based on the change in total strut length, disregarding local strains within the struts that potentially arise from bending behavior. Thirdly, the actual *in vivo* strain regimes may depend on many additional factors, including cage placement and patient-specific factors such as weight, bone density, degenerative state of the spine, and spinal curvature (Polikeit et al., 2003a; Abbushi et al., 2009; Naserkhaki et al., 2016; Galbusera et al., 2021). The influence of patient-specific variations on local strain regimes thus remains unknown.

Therefore, this study aimed to determine patient-specific variations in local strain patterns on the surface of a trussed titanium interbody fusion cage. Finite element (FE) modeling enables simulation of several physiological loading conditions and quantification of local strain values within spinal (sub) structures as well as within the cages (Goel et al., 2006; Gustafson et al., 2017). Additionally, the effect of patient-specific factors can be examined by studying the variation between different patient-specific models. Patient-specific FE models of four patients eligible for spinal fusion surgery were modified to simulate a posterior lumbar interbody fusion (PLIF) treatment with trussed titanium cages. Functional dynamics of the non-operated lumbar spinal segments, and the local cage strains and endplate stresses at the operated segment, were evaluated under physiological extension/flexion movement of the lumbar spine.

## Materials and Methods

### Patient-Specific FE Models of the Intact Lumbar Spine

Four patients were selected from a database of patients eligible for a spinal fusion operation as available from the earlier EU-funded MySpine project (EU FP7-ICT 269909). These patients were selected because they had similar vertebral sizes, such that the same cage design and size could be used in all patients, thereby excluding variation in the results due to differences in cage size. For all four patients, patient-specific FE models of the lumbar spine were available. The FE models were composed of the lumbar vertebrae (L1-L5), the IVDs (L1-2 to L5-S1), and the major ligaments per spinal motion segment. Detailed descriptions of patient data, model generation, and underlying material models can be found elsewhere (Malandrino et al., 2015; Rijsbergen et al., 2018) and are only described briefly here. Based on segmentations of vertebral structures via CT data and segmentations of IVD structures via magnetic resonance imaging (MRI) data, a generic FE model was morphed to patient-specific spinal geometries (Castro-Mateos et al., 2014; Castro-Mateos et al., 2015; Castro-Mateos et al., 2016).

Patient-specific trabecular bone densities were integrated in the models by defining transversely isotropic linear elastic material properties for each element, based on the mean CT gray value calculated within the representative volume of each element (Blanchard et al., 2016). Bony posterior elements, facets, and bony endplates were modeled as isotropic linear elastic materials, whereas the sacrum and cortical bone were modeled as orthotropic linear elastic materials. Surface articulation in the facet joints was assumed to be frictionless and resolved with a penalty normal stiffness of 200 N/mm (Schmidt et al., 2009). Cartilage endplates were modeled as isotropic poro-elastic materials, whereas the nucleus pulposus (NP) and annulus fibrosis (AF) were both modelled as poro-hyperelastic materials (Malandrino et al., 2014). The role of cross-ply collagen fibers present in the AF was implemented by adding an additional anisotropic term to the strain energy density function (Malandrino et al., 2013). Darcy’s law was used to determine the fluid pore pressure. The total stress in the poro-(hyper)elastic elements was defined as the sum of the fluid pore pressure and the porous solid stress as derived from the strain energy density function. An additional swelling pressure-related term was introduced for the NP to model proteoglycan-induced swelling of the IVD. Strain-dependent permeability was implemented and updated during the simulations for each poro-(hyper) elastic material model (Malandrino et al., 2015). Exact material parameters of the IVD substructures depended on the degenerative state of the IVD, which was previously determined by an experienced radiologist using the MRI data and the Pfirrmann grading system (Pfirrmann et al., 2001). The included ligaments were described as hypoelastic unidirectional materials of which the parameters differed per ligament type and disc level (Noailly et al., 2012). Pfirrmann grade-dependent material parameters for IVD substructures were optimized based on *ex vivo* creep tests of monosegments, and independent validation was achieved for the full L1-S1 patient-specific model thanks to *ex vivo* kinematic measurements (Malandrino et al., 2015). Supplemental material 1 provides a summary of the materials used within the FE models.

### Patient-Specific FE Models of the Operated Lumbar Spine

**FIGURE 1:**
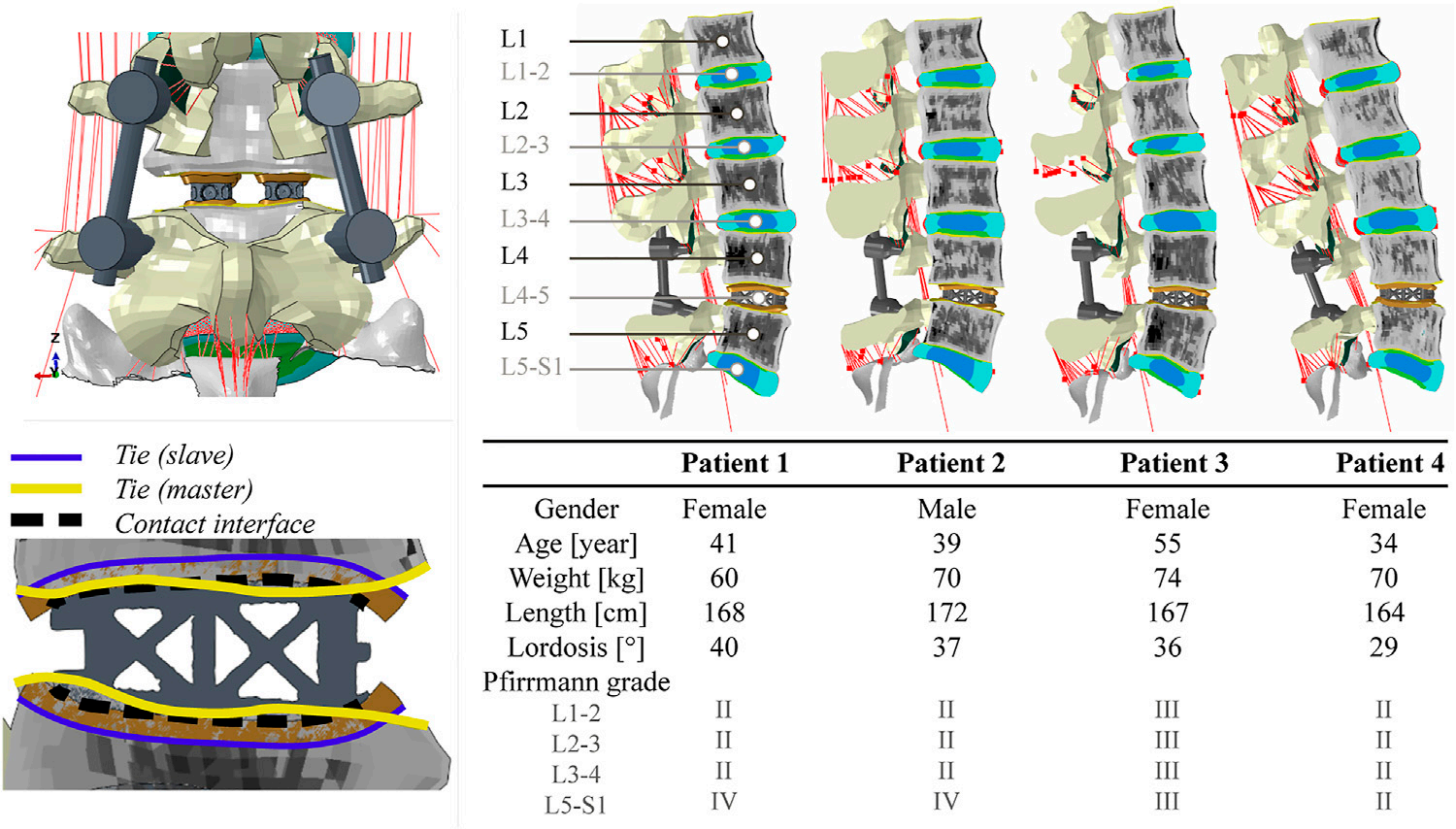
Top left: posterior view of segment L4-5 following PLIF surgery. A complete laminectomy was performed and two interbody cages were inserted. Bottom left: graphical overview of the interaction properties prescribed for cage vertebra interaction. The outer surface of the contact layer (purple) is rigidly tied to the associated bony endplate surface (yellow). Hard normal contact and a coefficient of friction of 0.20 were used to describe the contact between the cage and the inner surface of the contact layer (dashed black line). Right: a midsagittal cut of each of the four operated patient-specific models with corresponding demographic data. The different colors in the models represent different material properties.

Each of the four patient-specific FE models was modified to represent a situation directly after L4-5 PLIF surgery. A complete laminectomy was simulated which resulted in the removal of the elements of the spinous process and of all connecting ligaments at L4. In addition, the facet joints between L4 and L5, and the L4-5 IVD were virtually resected by eliminating the corresponding elements (Figure 1, top left).

In order to build the cage model to be implanted in each patient-specific model, a prototype trussed titanium PLIF cage was scanned at a 37 μm isotropic resolution in a microCT 100 system (SCANCO Medical AG, Brüttisellen, Switzerland) to retrieve the as-manufactured geometry of the cage. Scan data was imported into image processing software for design and modeling (Mimics Innovation Suite, Materialise, Leuven, Belgium). Following segmentation of the cage, a FE mesh was generated that consisted of 97,186 quadratic tetrahedral elements with a target triangle edge length of 0.30 mm to describe submillimeter details. Supplementary material 2 shows the geometry of the cage and how the meshing procedure affected the level of detail in surface features that was retained in the eventual cage models.

In order to accommodate interactive placement of the cages into the intervertebral disc space, without the need for laborious remeshing of the adjacent vertebrae, contact layers were introduced. Contact layers conforming to the top and bottom curvature of the cage were designed by using a computer aided design software (NX 12, Siemens PLM software, Plano TX, United States). These layers were 2.0 mm thick. They were imported in ABAQUS/Standard (Simulia, Inc., Providence, RI, United States) version 2018 and meshed, leading to approximately 25,000 linear brick elements per contact layer with a target triangle edge length of 0.33 mm. These layers, representing the cage endplate interface, were modeled as an isotropic linear elastic material with a Young’s modulus of 1,000 MPa and a Poisson’s ratio of 0.30 (Polikeit et al., 2003b). Although this stiffness value is believed to resemble the cage endplate interface appropriately, the exact stiffness depends on endplate preparation technique and might vary from the stiffness of cancellous up to cortical bone (100–10,000 MPa). To investigate the effect of these variations, a side study was performed (see Supplementary material 3).

To match the shape of the contact layers with the exterior struts of the cage, a deformable contact simulation was performed. Interaction between the inner surface of the contact layer and the interbody cage was modelled as hard normal contact with a coefficient of friction of 0.20 (Vadapalli et al., 2006). Then, the contact layers were moved 0.30 mm towards the cage and were allowed to deform, as the contact with the cage, modelled as a rigid body, was detected. The resulting deformed mesh of the contact layers was saved in its stress-free state, and the meshes of two interbody cages (one left and one right) and the corresponding contact layers were manually positioned within each patient-specific lumbar FE model to simulate an L4-5 interbody fusion. The outer surfaces of the contact layers were then rigidly tied to the bony endplate surface of the associated vertebra (Figure 1, bottom left). Because cage positioning is a manual procedure both in our models and in the clinic, the exact cage position can vary. To investigate the effect of these variations, a side study was performed (see Supplementary material 3).

Interbody cages were modelled as isotropic linear elastic titanium (Ti-6Al-4V, Young’s modulus of 116 GPa, Poisson’s ratio of 0.32). Finally, titanium (Ti-6Al-4V) pedicle screw and rod instrumentations were implemented in the models, based on anatomical landmarks of the spine. Pedicle screws (32 mm shaft length, 5 mm diameter) were fixed in the vertebrae and spinal rods (5 mm diameter) were fixed in the screw heads by embedding constraints. Figure 1 shows segment L4-5 following PLIF surgery and visualizes the imposed interaction properties between cage, contact layer, and vertebrae. In addition, the four operated models are visualized in this figure.

### Boundary and Loading Conditions

The caudal end of each lumbar spine model was completely constrained in all modeling steps. In the first step (8 h), the cranial end remained unconstrained allowing pre-swelling of the poro-(hyper)elastic IVD elements. In the second step (5 s), a patient-specific compressive load was applied to the spine by means of the follower load technique (Renner et al., 2007). Two node connector elements were placed bilaterally through the vertebral centers in the sagittal plane in order to apply a compressive load that is oriented tangent to the spinal curvature. Patient-specific magnitudes of the follower load (range 368–454 N) were based on previous literature (Han et al., 2013). In the third step (5 s), an extension or flexion movement was simulated. A total deflection of 20° was imposed at the cranial end of L1 while constraining all off-axis rotations. Simultaneously, the patient-specific follower load was set to increase during extension (range 748–888 N) and flexion (range 976 to 1,148 N). The patient-specific magnitudes of the follower load per loading condition were derived from the data of Han et al. by interpolating the literature values of the resultant force at spinal level L1 to the patient-specific weight and length characteristics of the patients included in this study.

### Output Analysis

ABAQUS/Standard (Simulia, Inc., Providence, RI, United States) version 2018 was used to solve extension and flexion simulations for each of the four patient-specific models. Load-deflection curves were determined for the complete lumbar spine and per non-operated functional spinal unit (FSU) as described before (Loenen et al., 2021). In addition, the intradiscal pressure (IDP) was quantified in the NP of the IVDs. It was defined as the superposition of the average pore pressure and the average axial component of the solid matrix stress. The absolute maximum principal strain values in the spinal cages were visualized and the percentage of surface nodes that exceeded an absolute strain value of 200 µε was quantified for each loading condition. Additionally, the normal contact pressures at the caudal cage-contact layer interface were visualized and the mean caudal contact pressure was quantified for each loading condition.

## Results

Figure 1 provides an overview of the demographic data of the four patients included in this study (one male and three female). As patients were selected to fit the same cage size, the population comprised a relatively narrow weight and length range (60–74 kg and 164–172 cm). Lumbar lordotic angles ranged from 29 to 40° while degenerative state of the non-operated discs varied from Pfirrmann grade II to IV. Different gray value intensities and distributions in the trabecular bone regions indicate the differences in bone density between vertebrae and patients.

**FIGURE 2:**
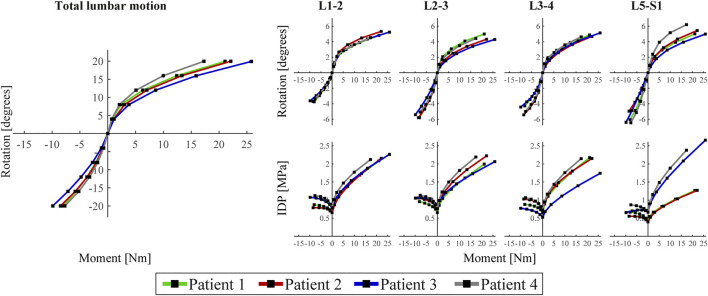
Left: Load-deflection curve of the total lumbar spine for the four patient-specific models. Rotation represents rotation of the cranial endplate of L1 in the sagittal plane and moment is the reaction moment required to obtain these rotation values. Right: angular motion per functional spinal unit (FSU) and intradiscal pressure (IDP) per intervertebral disc (IVD) of the unoperated levels of each of the four patients. For all (sub)figures, negative and positive moments/rotations describe extension and flexion, respectively.

Figure 2 displays the total lumbar spine motion, the angular motion per FSU, and the IDP per IVD during extension/flexion movement. The four S1-L1 patient-specific models showed comparable asymmetrical extension/flexion flexibility profiles but differed in terms of reaction moment magnitudes at 20°, in both extension (range −9.9 to −7.7 Nm) and flexion (range 17.3–25.8 Nm). These patient-specific differences were also reflected in the angular motion per FSU, especially in flexion at L5-S1. L5-S1 was also the disc with most patient-to-patient variability in terms of degenerative state (Figure 1). The IDP over all discs in neutral position, under follower load compression, ranged from 0.4 to 0.8 MPa. In general, it increased more in flexion (range 1.3–2.7 MPa) than in extension (range 0.6–1.1 MPa). Again, patient-specific variations were most pronounced in flexion at the L5-S1 level. The Pfirrmann grade III L3-4 IVD model (patient 3) led to clearly lower IDP values than the grade II L3-4 IVD models of the other patients, during the flexion movement.

**FIGURE 3:**
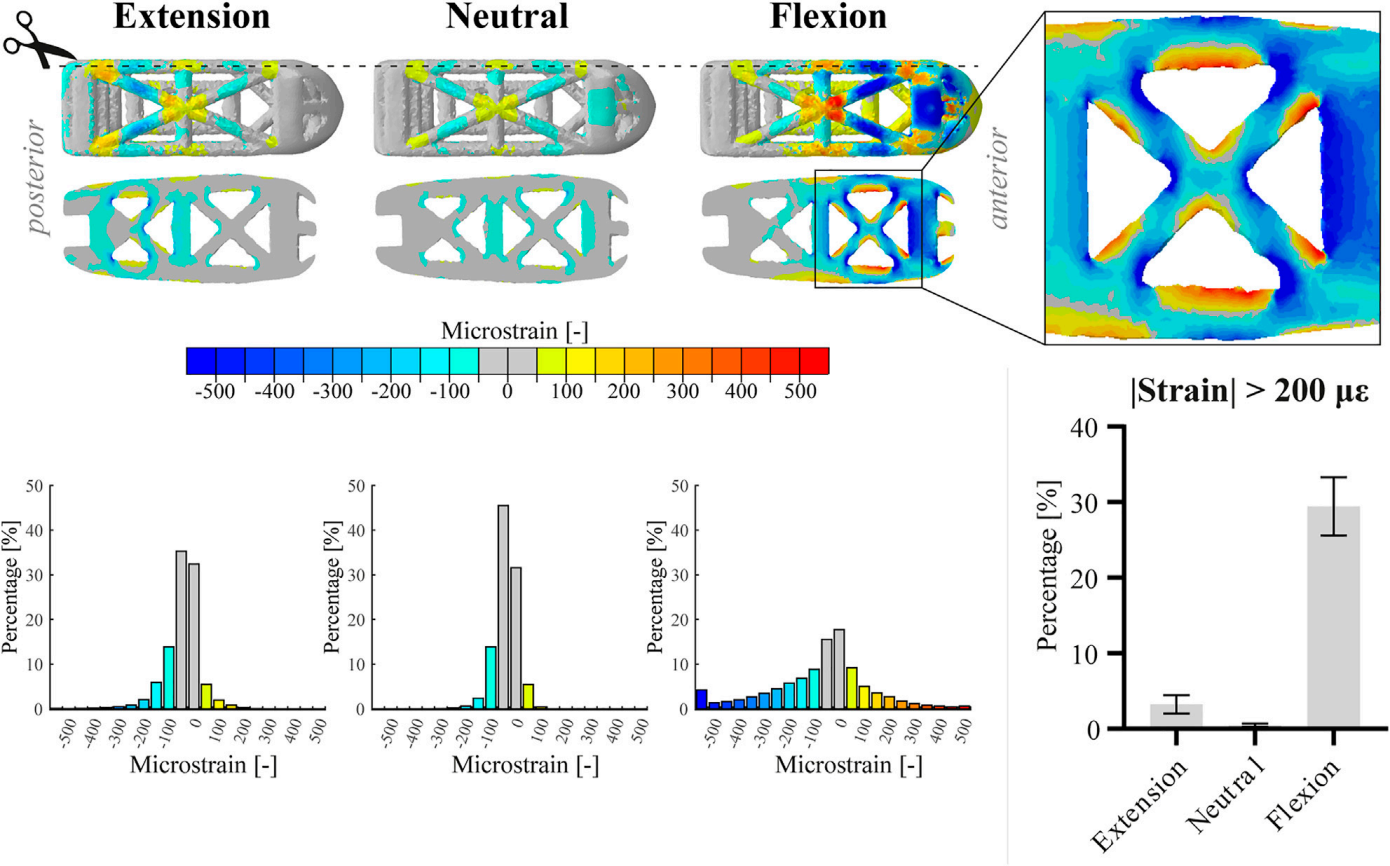
Top part of the figure shows the absolute maximum principal strain values (top view and sagittal cut, respectively) on the right cage of patient 1 in extension, neutral, and flexion position. Additionally, an enlarged view of the anterior part of the cage in flexion is displayed. The three histograms correspond to the images above and represent the relative amount of surface nodes [%] for different strain ranges. For the bar chart, data of both cages within one patient were amalgamated. The bar chart displays the relative amount of surface nodes [%] exceeding an absolute strain value of 200 µε for the different loading conditions. Bars represent the mean and 95% confidence interval of the patient population (n = 4).

Since the two inserted PLIF cages (left, right) demonstrated similar deformations within one patient for each of the loading conditions, Figure 3 illustrates only the calculation outcomes in the right cage of patient 1. In neutral position, only small strain values (<200 µε) were calculated in the cage. In extension, strains shifted to the posterior side of the cage and values increased up to approximately 300 µε. In flexion, strains shifted to the anterior side of the cage and locally, values exceeded 500 µε. For all loading conditions, both compressive (negative) and tensile (positive) strains were present at the struts. In the enlarged inset, struts show compressive strain on the one side and tensile strain on the other side, indicating inwards bending of the struts during flexion. The bar chart demonstrates that the relative amount of surface exceeding 200 µε varied more between loading conditions than between patients, whereas the coefficient of variation in flexion was 8.3%. The peak von Mises stresses within the PLIF cages ranged from 248 to 304 MPa over the different patients, which is far below the yield stress of 3D printed titanium.

**FIGURE 4:**
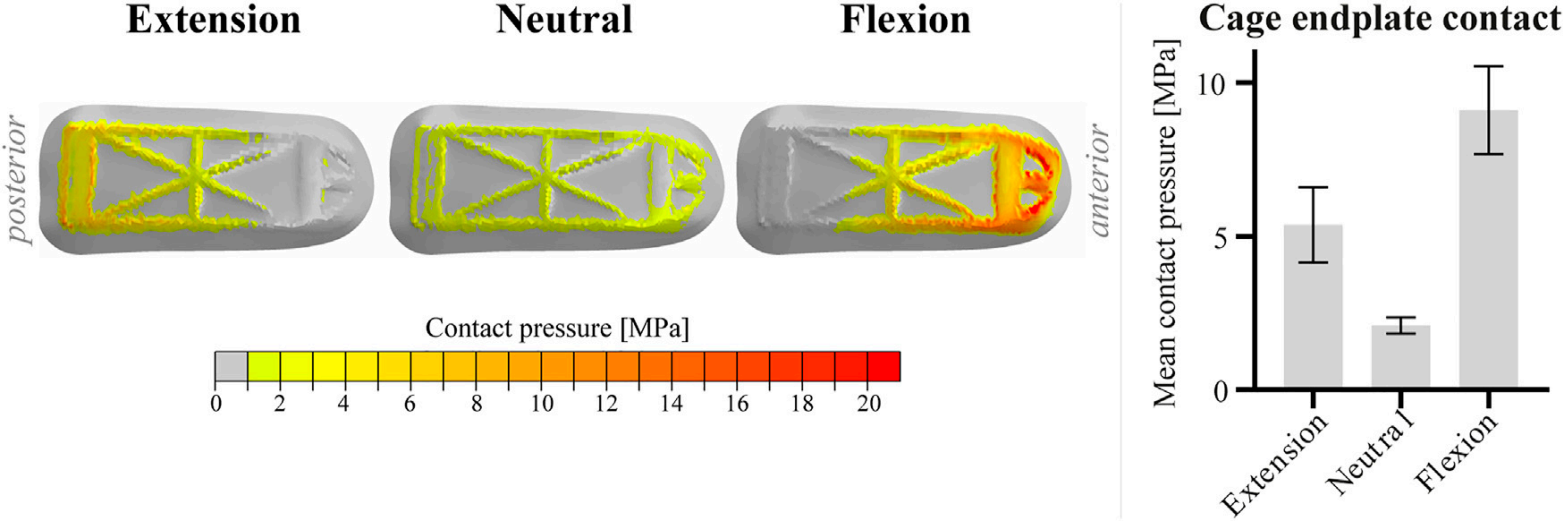
Visualization of the contact pressure of the right cage of patient 1 on the caudal contact layer in extension, neutral and flexion position. For the bar chart, data of the two caudal contact layers within one patient were amalgamated. The bar chart displays the mean caudal contact pressure for the different loading conditions. Bars represent the mean and 95% confidence interval of the patient population (n = 4).

Since the two caudal contact layers (left, right) within one patient showed similar behavior for each of the loading conditions, Figure 4 shows only the graphical output at the right caudal contact layer of patient 1. Comparable to the strain distribution in the cages, the caudal contact pressure shifts posteriorly and anteriorly in extension and flexion, respectively. Highest caudal contact pressures were observed anteriorly in the flexion configuration. Like the relative amount of surface exceeding 200 µε, the mean caudal contact pressure varied more between loading conditions than between patients (see bar chart). The coefficient of variation of the mean caudal contact pressure in flexion was 9.9% between patients.

## Discussion

The main purpose of this study was to determine patient-specific variations in local strain patterns on the surface of trussed titanium interbody fusion cages. These variations were analyzed in a specific subgroup allowing for implementation of one specific PLIF cage size for all included patients. The results demonstrate that within this specific subgroup patient-specific factors such as weight, bone density, degenerative state of the spine, and spinal curvature did affect local strain regimes; however, loading conditions in this group had a much more prominent impact on both size and distribution of the strains. The same trend was observed for the mean contact pressure of the cages at the caudal vertebral endplates. It should however be emphasized that the patient dataset in current research did not include any patients with comorbidities like previous lumbar surgery, heavy smoking, drug use or other conditions affecting bone or disc metabolism, osteoporosis, obesity, or scoliosis. Therefore, this patient cohort does not reflect the broad patient population undergoing spinal fusion surgery. Inclusion of patients with these comorbidities could provide additional insight as to what patient-specific constraints need to be taken into consideration and how to optimize implant design to address these conditions.

Although the lumbar spine models used in this research were validated in earlier studies, further validation would be warranted, particularly because the earlier studies did not include the instrumentation modeled in this research. A logical next step would be to validate the predicted strains within the cages using *ex vivo* spine testing of operated spines such that the FE results can be verified against the *ex vivo* observations. Subsequently, a patient study in which strain results, obtained from patient-specific models, are correlated with the postoperative progression of bone growth would provide further clinical evidence.

The operated patient-specific lumbar spine models in this study are somewhat stiff in flexion and somewhat compliant in extension (Panjabi et al., 1994; Dreischarf et al., 2014). This behavior might be caused by the surgical modifications to the intact models, as spinal fusion surgery generally increases the stiffness of the spine more in flexion than in extension movement (Molz et al., 2003). Since the rotational contribution of FSU L4-5 is known to be proportionally larger in flexion compared to extension (Pearcy et al., 1984), this effect might have been enhanced as all patients were scheduled for L4-5 interbody fusion. Additionally, the implementation of the follower load might have had a minor effect on angular motion per FSU (Rohlmann et al., 2001). The differences observed between patients on total lumbar motion level mainly emerged from FSU L5-S1, whose degenerative state varied most substantially between patients (range II to IV on the Pfirrmann grading system). Because the reduced disc height of a degenerated disc presumably increases the stiffness of the FSU (Muriuki et al., 2016), different load-deflection curves between segments could be expected. Mean IDP values in neutral position were consistent with *in vivo* data and increased, in accordance with previous literature, more substantially in flexion than in extension (Wilke et al., 2001). As IDP is known to increase more significantly in flexion in the fused spine compared to the native spine (Weinhoffer et al., 1995), IDP values were found to be in the high-end regime. The differences in IDP observed for disc L3-4 and L5-S1 in flexion correspond with loss of water in the more degenerated discs, inducing lower IDP values (Sato et al., 1999). Overall, all patient-specific models revealed physiologically realistic functional dynamics of the operated spine.

The percentage of the cage that experienced strains consistent with those reported to maintain bone balance under physiological loading in the current study were slightly less than those found in the aforementioned study that investigated a different trussed titanium cage under moderate (1,000 N) axial compression (Caffrey et al., 2016), i.e., up to 50% of the free struts was loaded beyond 200 µε in the *ex vivo* study versus up to 30% of the total surface in current *in silico* approach. These differences, however, might be explained by the fact that in the *ex vivo* study a larger cage was used, which has a relatively smaller screw insertion block. In the prototype PLIF cage of the current study the screw insertion block carries a relatively large part of the load, thereby reducing the load on the struts. Therefore, it can be expected that for a similar cage design the actual strain values would compare well to those reported in the earlier study.

In current research, a value of 200 µε was adopted in order to quantify the percentage of surface that experienced a strain value relevant for preserving bone homeostasis and stimulating bone formation. It has however been described before that the exact strain threshold for maintaining bone mass is a nonlinear function of the daily loading cycle number (Rubin et al., 2001). Stimuli with magnitudes of 200 µε are estimated to require approximately 35,000 loading cycles per day (once every 2–3 s) to maintain bone mass, whereas for mechanical stimuli with a frequency in the order of 10^6^ to 10^7^ per day (10–100 cycles per second) even strain values lower than 10 µε are suggested to be capable of stimulating bone formation (Qin et al., 1998). Patient-specific spinal motion profiles may therefore be required to interpret the strain values more appropriately. Moreover, it is worthwhile to emphasize that these reference strain values originate from bone remodeling research and it is unknown to what extent these values can be directly translated towards a former intervertebral disc, i.e., a cartilaginous environment. Once interbody fusion has progressed between the vertebrae, these values would be directly applicable. This would, however, require extension of the FE models to include the formation of bone within the cages and was outside the scope of this research.

Although the FE models were intended to predict strain values at the surface of trussed cages on submillimeter scale, they do not provide a full characterization of the mechanical stimuli the attached cells might perceive in an *in vivo* situation. This is because the exact micro-to nanoscale surface features at the struts, the way cells could be attached to the struts (bridged versus non-bridged), and other mechanical stimuli like fluid flow and hydrostatic pressure in the cages were not involved in current FE models (Kapur et al., 2003; Zhao et al., 2015; Hasegawa et al., 2020). Also, the trussed titanium cage model did not take surface micro-to nanostructure and strut composition in consideration. This simplified cage model therefore provides only a limited representation of the cage properties regarding its *in vivo* mechanobiological response. In order to accurately represent this response, multi-scale modelling will be required including microstructural features that include cell-strut interaction and fluid flow within the cage.

Since the posterior side of the PLIF cage is shielded more by the pedicle screw and rod instrumentation than the anterior side, higher strains could be found in the anterior part of the cage under extension/flexion movement. Assuming the higher strains will indeed accelerate bone formation at the anterior side of the cage, this would be favorable from a biomechanical point of view as PLIF segments that are partially fused anteriorly are found to be more stable than those partially fused posteriorly (Bono et al., 2007).

Current research used a subset of patient-specific FE models to predict the impact of patient-specific factors on cage level. The same framework could also serve as a platform to evaluate several design modifications of the interbody cages iteratively. Design modifications might be considered in order to target higher surface strains or to distribute the strains more homogenously across the whole cage. However, the ultimate strength of the proposed design modifications should also be continuously monitored as interbody cages should also withstand high peak forces in the lumbar spine (Ledet et al., 2018). Additionally, modified designs could change the amount of direct cage to endplate contact thus affecting the stresses on the endplate and the risk for cage subsidence (Steffen et al., 2000). Cage design optimization algorithms would therefore require a cost function that assesses a combination of several output metrics. In the future, development of such algorithms may facilitate interbody cage design optimizations.

It should be noted that the current study analyzed the behavior of one specific trussed titanium cage geometry used for LIF treatment with a posterior approach (PLIF cage) and that results might be different for other cage geometries. In fact, the choice for another surgical approach, like anterior lumbar interbody fusion (ALIF), would affect the output on the cage level by multiple means. ALIF surgery requires only one large cage as the anterior approach provides full access to the ventral side of the operated spinal segment (Mobbs et al., 2015). Since each single cage contains a screw anchoring point to enable cage insertion during surgery, one trussed ALIF cage contains relatively more struts than two trussed PLIF cages. Additionally, one ALIF cage generally comprehends a larger footprint on the vertebral endplate than two PLIF cages do. Moreover, ALIF surgery can be performed as stand-alone procedure, which generally means there is some additional fixation that can be instrumented anteriorly directly after cage placement (e.g., an anterior fixating plate), but there is no pedicle screw and rod instrumentation or other supplemental posterior fixation involved (Manzur et al., 2019). The different types of additional fixation in PLIF and ALIF surgery obviously result in different loading patterns on the interbody fusion construct (Choi et al., 2013).

In conclusion, this study demonstrates the applicability of patient-specific FE models to quantify the impact of patient-specific factors as weight, bone density, degenerative state of the spine, and spinal curvature on interbody cage loading. As the resulting surface strains were very similar for the different patient-specific models in the selected patient group, it can be concluded that the trussed design is rather robust from a mechanobiological perspective. In the future, the same framework might be further developed in order to establish a pipeline for interbody cage design optimizations.

## Data Availability

The datasets presented in this article are not readily available because the data is kept at the different institutes according to project regulations and the institutions involved follow national regulations with regard to data. Requests to access the datasets should be directed to BvR, b.v.Rietbergen@tue.nl.
