# Supplementary material for: Patient-Specific Variations in Local Strain Patterns on the Surface of a Trussed Titanium Interbody Cage
Source: Front Bioeng Biotechnol. 2022 Jan 11;9:750246. doi: 10.3389/fbioe.2021.750246 (PMC8786731; doi:10.3389/fbioe.2021.750246)
Supplement: Supplementary file 1 [file DataSheet1.PDF]

SUPPLEMENTARY MATERIAL 1 | Summary of the materials used within the models

Component	Element type	Constitutive law	Parameters
Trabecular bone	C3D8	Transversely isotropic linear elastic	Young's moduli and Poisson's ratios based on mean CT gray value calculated within the representative volume of each element
Cortical bone	C3D8	Orthotropic linear elastic	E_1 = 8,000 MPa<br>E_2 = 8,000 MPa<br>E_3 = 12,000 MPa<br>ν_{12} = 0.4<br>ν_{13} = 0.35<br>ν_{23} = 0.3<br>G_{12} = 2,000 MPa<br>G_{13} = 2,400 MPa<br>G_{23} = 2,400 MPa
Bony posterior elements	C3D8	Isotropic linear elastic	E = 3,500 MPa<br>ν = 0.3
Facet cartilage	C3D8	Isotropic linear elastic	E = 20 MPa<br>ν = 0.4
Bony endplate	C3D8	Isotropic linear elastic	E = 1000 MPa<br>ν = 0.3
Cartilage endplate	C3D8P	Poro-elastic:<br>• Isotropic linear elastic solid<br>• Fluid pore pressure derived from Darcy's law	 • E = 20 MPa ν = 0.17<br>• Strain dependent porosity and permeability
Annulus fibrosis	C3D8P	Poro-hyperelastic<br>• Isotropic hyperelastic solid including anisotropic fibers<br>• Fluid pore pressure derived from Darcy's law	 • Shear and bulk modulus, as well as fiber organization depend on the Pfirrmann grade<br>• Strain-dependent porosity and permeability. Initial porosity and permeability values depend on the Pfirrmann grade
Nucleus pulposus	C3D8P	Poro-hyperelastic:<br>• Isotropic hyperelastic solid<br>• Fluid pore pressure derived from Darcy's law, assuming constant presence of a swelling pressure related term	 • Shear and bulk modulus depend on the Pfirrmann grade<br>• Strain-dependent porosity and permeability. Initial porosity and permeability, as well as the swelling pressure related term value depend on the Pfirrmann grade
Ligaments	T3D2	Unidirectional hypo-elastic	Parameters differ per ligament type and spinal motion segment
Titanium	C3D8	Isotropic linear elastic	E = 116,000 MPa<br>ν = 0.3

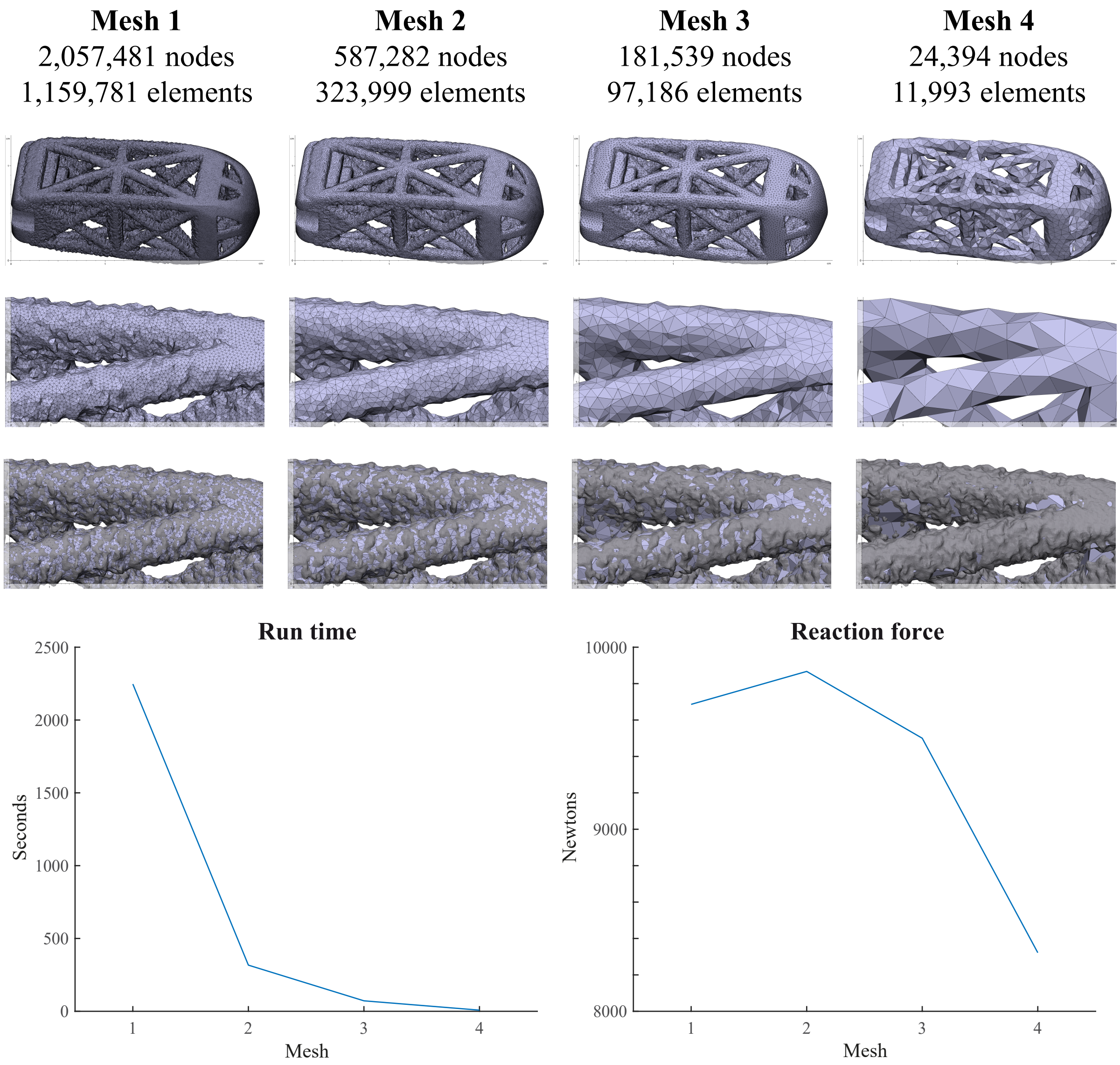

SUPPLEMENTARY MATERIAL 2 | Mesh refinements of the prototype trussed titanium PLIF cage that was used in the current study. For each mesh, the mesh of the whole cage (first row) as well as a zoomed in area (second row) is shown. In the third row, the geometry of the cage as derived by segmentation of the 37 micrometer isotropic resolution micro computed tomography scan is added to indicate the level of detail that was retained in each of the meshes of the cage. The graphs below the meshes show the run time and total reaction force across the cage meshes for a 0.1% compression analysis. Based on this output, the third mesh was eventually used in the patient-specific models.

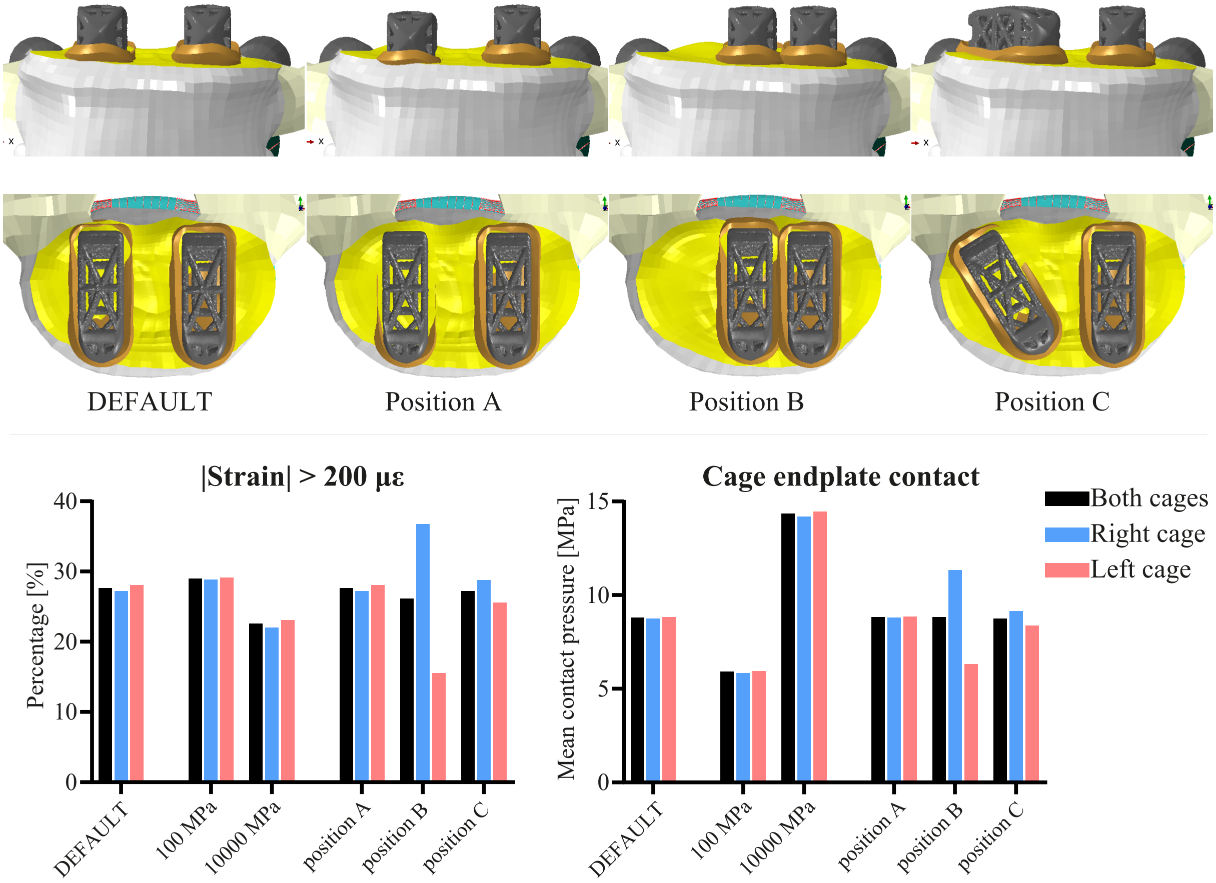

SUPPLEMENTARY MATERIAL 3 | Overview of the side study about the effect of variation in contact layer stiffness and cage positioning. At the top of the image, the default and three alternative cage positions are graphically shown. In position A, the right cage is translated 1.2 mm caudally. In position B, the right cage is translated 8.6 mm medially. In position C, the right cage is rotated 30 degrees in the axial plane. For all positions, the left cage remained at the default position. The stiffness and positioning variations were implemented for patient 1 and strains and stresses were evaluated in flexion movement only. The left bar chart shows the percentage of surface nodes that exceeded an absolute strain value of 200 με and the right bar chart shows the mean contact pressure at the caudal cage-contact layer interface. For both charts, data is presented for the left and right cage separately, as well as combined. Changing the stiffness of the contact layers did not induce different strain or stress output between the left and right cage but did result in different absolute values. These differences can be mainly found for the mean contact pressure. A stiffer contact layer is less forgiving and thus results in local peak forces without further distribution of the loads. Consequently, the contact pressure increases and less surface nodes experience a strain above the 200 με. The maximum strain experienced by surface nodes, however, does increase as result of these high local forces. Variation in cage positioning could induce differences between the left and right cage while the combined output remained unchanged. Asymmetric positioning may lead to asymmetric loading of the cages explaining the variations in strain and stress output are most pronounced for position B.
